# Application of machine learning in prediction of Chemotherapy resistant of Ovarian Cancer based on Gut Microbiota

**DOI:** 10.7150/jca.46621

**Published:** 2021-03-15

**Authors:** Ting-Ting Gong, Xin-Hui He, Song Gao, Qi-Jun Wu

**Affiliations:** 1 Department of Obstetrics and Gynecology, Shengjing Hospital of China Medical University, Shenyang, China.; 2 Department of Clinical Epidemiology, Shengjing Hospital of China Medical University, Shenyang, China.

**Keywords:** gut microbiota, ovarian cancer, chemoresistant, machine learning, random forest

## Abstract

**Background:** Ovarian cancer (OC) has the highest mortality among gynecological malignancies, and resistance to chemotherapy drugs is common. We aim to develop a machine learning approach based on gut microbiota to predict the chemotherapy resistance of OC.

**Methods:** The study included patients diagnosed with OC by pathology and treated with platinum and paclitaxel in Shengjing Hospital of China Medical University between 2017 and 2018. Fecal samples were collected from patients, and 16S rRNA sequencing was used to analyze the differences in gut microbiota between OC patients with and without chemotherapy resistance. Nine machine learning classifiers were used to derive the chemotherapy resistance of OC from gut microbiota.

**Results:** A total of 77 chemoresistant OC patients and 97 chemosensitive OC patients were enrolled. The gut microbiota diversity was higher in OC patients with chemotherapy resistance. There were statistically significant differences between the two groups in Shannon indexes (P <0.05) and Simpson indexes (P <0.05). Machine learning techniques can predict the chemoresistance of OC, and the random forest showed the best performance among all models. The area under the ROC curve for RF model was 0.909.

**Conclusions:** The diversity of gut microbiota was higher in OC patients with chemotherapy resistance. Further studies are warranted to validate our findings based on machine learning techniques.

## Introduction

Ovarian cancer (OC) is the leading cause of mortality among gynecologic malignancies worldwide, although the burden estimates (e.g. incidence, prevalence, and mortality) of this disease show a decrease in recent decades 1,2. Because of the lack of specific symptoms, most OC patients are diagnosed at late stages 3. Platinum and paclitaxel are currently the first-line chemotherapy regimens for OC. However, despite advances in treatment modalities, the prognosis of patients with OC remains poor. Approximately 20% to 30% of OC patients show recurrence or disease progression within 6 months after chemotherapy, and the median overall survival is approximately 12-18 months, requiring repeated systemic treatment 1. Additionally, intrinsic resistance occurs in approximately 15-25% of patients with OC, and almost all patients with recurrent disease ultimately develop platinum resistance, which can result in death 5,6. Chemotherapy resistance is related to multiple mechanisms such as alterations in the transport and cellular turnover of the drug and nonspecific cytoplasmic defense systems and DNA repair molecular systems 7. Many studies have looked into resistance mechanisms in the past and have provided insight into platinum resistance.

The intestinal microbiome plays a crucial role in modulating the risk of several chronic diseases, including obesity 8,9, cardiovascular disease 10, metabolic abnormalities 11, and cancer 12. The delicate balance of the intestinal microbiome composition is essential for maintaining intestinal immunity and whole-body homeostasis 13. Gut microbiota imbalance is closely related to the occurrence of cancers 14,15. Imbalance of the intestinal microbiota can be transferred to adjoining organs or even to distant organs through the blood 13. For example, the gut microbiota can affect the liver, activating the immune system and causing necrosis and apoptosis of liver cells, which can lead to the formation of severe fibrosis and even liver cancer 16. A recent report showed that *Fusobacterium* nucleatum promotes chemoresistance in colorectal cancer by inducing autophagy and selective loss of miR-18a* and miR-4802 in cancer cells 17.

In OC, Chase et al. published a review and demonstrated that the microbiota is linked to OC initiation and progression through effects on intestinal inflammation and tumor-related signaling pathways 18. A recent study showed that the microbial composition and its alterations are associated with OC, as the diversity and richness indexes were significantly decreased in OC tissues 19. The ratio of the two phyla for Proteobacteria/Firmicutes is increased in OC, and a set of genes named human antibacterial-response genes that include inflammation-associated or immune-associated genes are involved in OC initiation and progression 19. However, the relationship between the gut microbiota and chemotherapy resistance in OC patients has not been investigated to date.

Unlike traditional prediction models (e.g., logistic regression), modern machine learning can automatically learn the underlying patterns of data without any implicit assumptions. It has become an alternative method for developing prediction models 20. In this study, we firstly use 16S rRNA sequencing technology to characterize the overall structure of gut microbiota in chemoresistant and chemosensitive OC patients. Furthermore, machine learning is used to find the key microbiota that can predict the chemotherapy response of OC.

## Materials and methods

### Patient enrollment and sample collection

This study was a case-control study conducted at Shengjing Hospital of China Medical University (Shenyang, China) between 2017 and 2018. The inclusion criteria were patients aged 18-75 years who were diagnosed with OC by pathology and who had complete medical records. All enrolled patients received optimally and maximally cytoreduced surgery. And all patients took carboplatin and taxol as the primary chemotherapy regimens, no other chemotherapy drugs such as bevacizumab were used. According to the 2016 clinical practice guidelines in OC published by the National Comprehensive Cancer Network, chemotherapy resistant was defined as an initial response to chemotherapy followed by relapse within 6 months of the completion of chemotherapy 21. Chemotherapy sensitive was defined as a clear response to chemotherapy treatment and relapsed after more than 6 months of the completion of chemotherapy treatment. Subjects who had taken antibiotics or probiotics in the past 1 month were excluded. Subjects who receiving neoadjuvant chemotherapy were also excluded. The above information was obtained from medical records. Chemoresistant OC patients were selected as the case group, and chemosensitive patients matched by age, International Federation of Gynecology and Obstetrics (FIGO) stage, and pathological classification were selected as the control group. This study was approved by the Ethics Committee of Shengjing Hospital of China Medical University (2015PS38K). The clinical information of this study was extracted from the electronic medical record system of Shengjing Hospital of China Medical University, fully protect the privacy of patients. Stool samples were collected after routine diagnosis and treatment in clinic, which did no harm to patients.

### Sample collection, DNA extraction, and 16S rRNA gene amplification sequencing

Fecal samples were collected at the hospital before each chemotherapy. Fresh fecal samples were collected from enrolled patients after natural defection in the clean toilet, using the scoop in the collection tube take 3-5 g fecal and place in the collection tube. Then used sterilized 2.5 mL tubes containing pure ethanol and frozen at -80 °C until DNA extraction 22. Total DNA from fecal samples was extracted using the Cetyltrimethylammonium bromide method 23. DNA concentration and purity were monitored on 1% agarose gels. According to the concentration, DNA was diluted to 1 ng/μL using sterile water. Amplification and sequencing of the V4 hypervariable region of the 16S rRNA gene was performed using the validated, region-specific bacterial primers 515F (5′-GTGCCAGCMGCCGCGGTAA-3′) and 806R (5′-GGACTACHVGGGTWTCTAAT-3′) 24. 16S rRNA genes were amplified used the specific primer with the barcode. All PCR reactions were carried out in 30 μL reactions with 15 μL of Phusion® High-Fidelity PCR Master Mix (New England Biolabs), 0.2 μM of forward and reverse primers, and 10 ng template DNA. Thermal cycling consisted of initial denaturation at 98 °C for 1 min, followed by 30 cycles of denaturation at 98 °C for 10 s, annealing at 50 °C for 30 s, and elongation at 72 °C for 30 s, with a final step at 72 °C for 5 min. Amplified products from fecal samples were verified by gel electrophoresis using 5 mL of the PCR reaction mixture in a 2% agarose gel. The PCR products were purified using the GeneJET Gel Extraction Kit (Thermo Scientific). Sequencing libraries were generated using NEB Next® Ultra™ DNA Library Prep Kit for Illumina (NEB, USA) following the manufacturer's recommendations, and index codes were added. The library quality was assessed on the Qubit@ 2.0 Fluorometer (Thermo Scientific) and Agilent Bioanalyzer 2100 system. The library was sequenced on an Illumina HiSeq platform and 250 bp paired-end reads were generated.

### Analysis of 16S rRNA gene sequences

Sequences were analyzed using the Quantitative Insights Into Microbial Ecology (QIIME) software package 25, and in-house Perl scripts were used to analyze alpha-(within samples) and beta-(among samples) diversity. First, reads were filtered by QIIME quality filters. Then, pick_de_novo_otus.py was used to pick operational taxonomic units (OTUs) by generating an OTU table. Sequences with ≥ 97% similarity were assigned to the same OTUs. Representative sequences for each OTU were selected, and the RDP classifier was used to annotate taxonomic information for each representative sequence 26. The OTUs that reached a 97-nucleotide similarity level were used for alpha diversity (Shannon, Simpson index) and richness analysis (ACE and Chao1, respectively). Rarefaction curves and Rank-Abundance curve were generated based on these three metrics.

### Construction of gut bacterial database and data preprocessing

The main purpose of this study was to develop a machine learning approach based on gut microbiota. The relative abundance of gut microbiota was used to construct the database. Those unclear gut microbiota were eliminated. Since the relative abundance value of all data is too small, -log10 is used for processing, and the missing value is treated as -log10 (1e-10).

### Supervised machine learning classifiers

In this study, nine types of supervised machine learning classifiers, including Naive Bayes (NB), Generalized Linear Model (GLM), Logistic Regression (LR), Fast Large Margin (FLM), Deep Learn (DL), Decision Tree (DT), Random Forest (RF), Gradient Boosted Trees (GBT), and Support Vector Machine (SVM) were assessed 27. All machine learning classifiers were implemented using RapidMiner (9.5.001) with the default parameters.

### Random Forest model classifier

**Figure 1:**
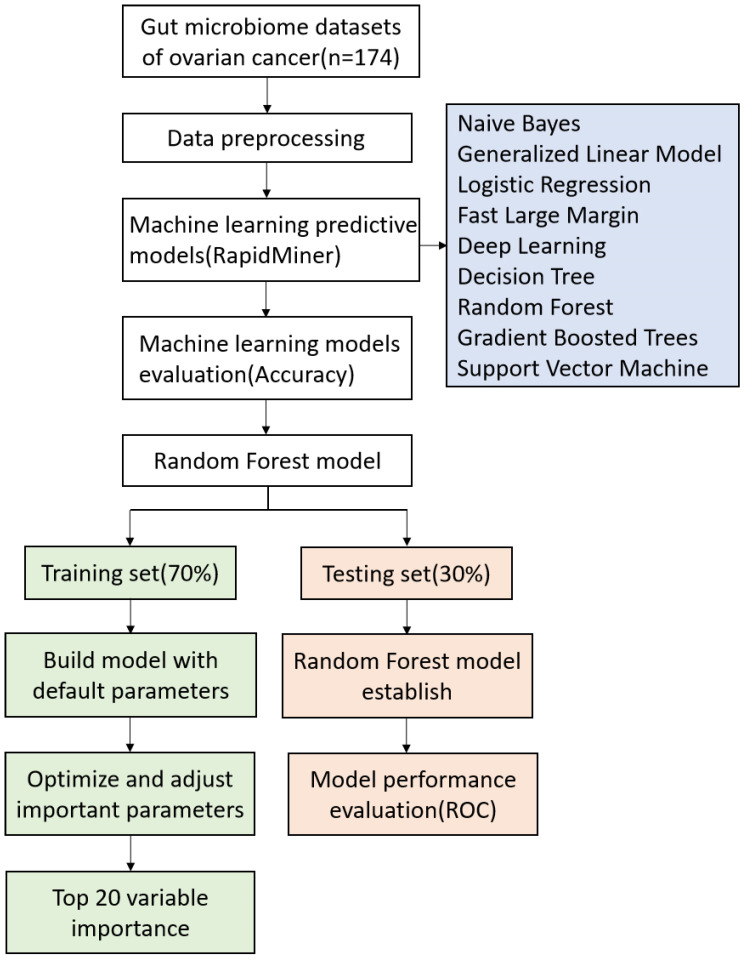
The working process of machine learning and random forest model.

The random forest (RF) was implemented using the randomforest package in R (http://cran.r-project.org//). RF classification is an ensemble learning method widely used for classification of large data 28. Random forest classification algorithm is based on the constructing multiple decision trees according to the bagging method: each tree is constructed independently from a bootstrap sample of the entire dataset 29. To avoid overfitting, each decision point, is split using the best abundance threshold in the randomly selected prediction subset, which based on the Gini criterion 29. At RF model, parameters such as mtry, nodes, trees, and node size have a significant impact on the performance of the model. The optimum parameters were carried out using the tuneRF fuction present in the RF package of R 30. Using the method of random sampling, 70% of the samples were used as training set to build the model, and the remaining 30% were used as test set to evaluate the model performance. Classifiers were trained using repeated 5-fold cross-validation of training dataset, and predictive performance was evaluated in the test dataset 31. Workflow for prediction of chemotherapy resistance in OC using machine learning methods and detailed RF model are presented in Fig. 1.

## Results

### Study population

A total of 174 OC patients were enrolled, of which 77 were resistance to chemotherapy and 97 were not. The characteristics of the participants are shown in Table 1. The baseline characteristics of the two groups showed no differences in age, FIGO stage, and pathological typing (P > 0.05).

### Gut microbiota structure in OC patients

**Figure 2:**
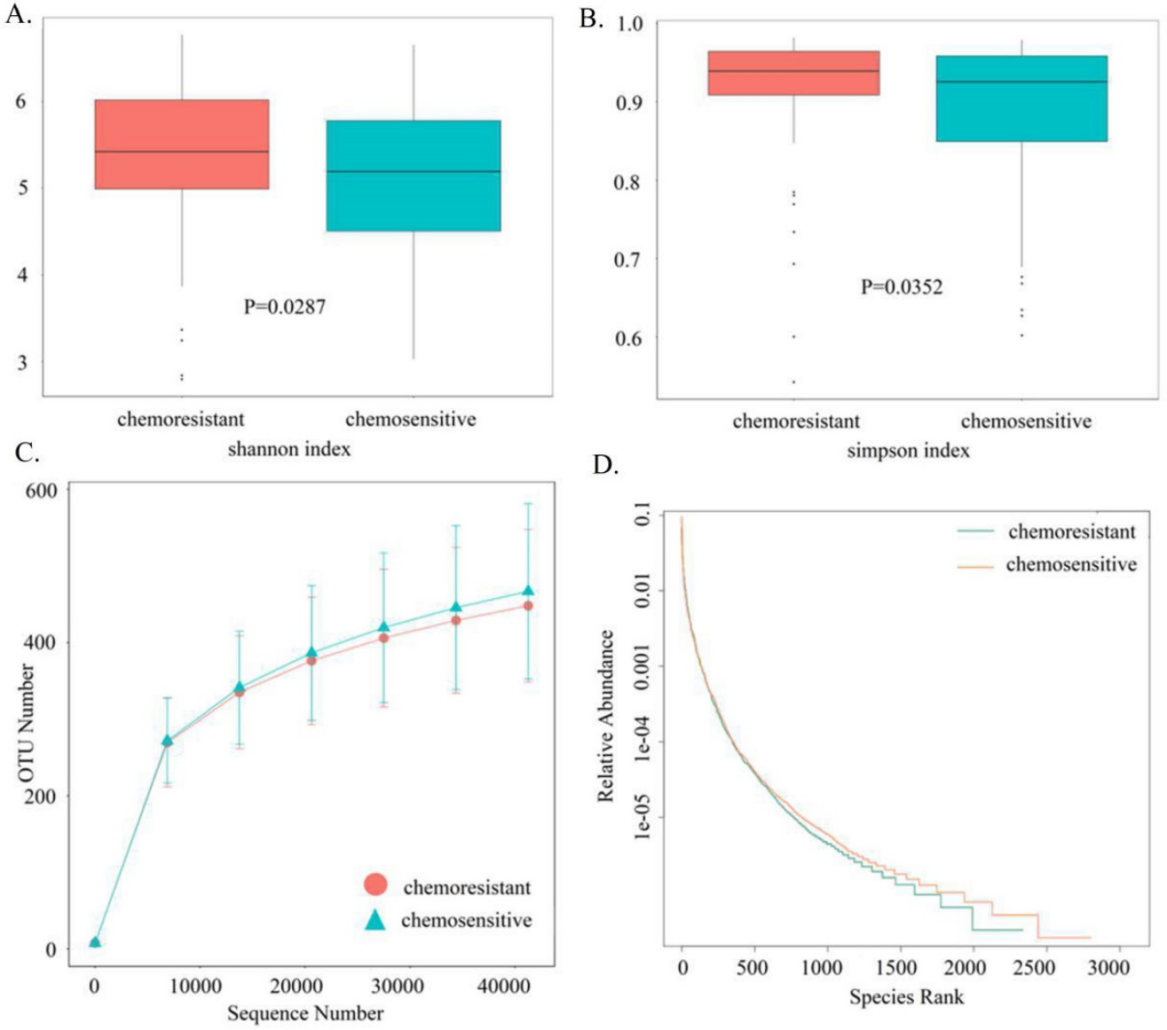
Comparison of the gut microbiota structures between chemoresistant and chemosensitive ovarian cancer patients. (A) Shannon index (B) Simpson index (C) Rarefaction curves (D) Rank-Abundance Curve.

Based on sequencing platform, single-end sequencing method was used to construct small fragment library for single-end sequencing. Through Reads shearing and filtering, 73,170 Reads were measured on average per sample, and 69,440 effective data were obtained on average after quality control, with the quality control effective rate reaching 94.96%. To characterize the gut microbiota structure in OC patients with or without chemotherapy resistance, the estimators of OTU number, coverage percentage, community richness index (Chao1 and ACE), and diversity index (Shannon and Simpsons) were compared between the two groups (Table 2). With 97% Identity, sequence clustering is transformed into Operational Taxonomic Units, and a total of 3,049 OTUs are obtained. And 2049 of 3,049 OTUs were shared between the two groups, accounting for 67.2% of the total richness. There were 273 OC patients with chemotherapy resistance specific-species and 727 patients without chemotherapy resistance specific-species. Good's coverage approached 99.8%, indicating that the sequencing depth of intestinal microbiomes was reasonable. Adonis testing showed that the evenness of the groups was significantly different (R^2^ = 0.01002, P < 0.05). There were statistically significant differences between the two groups in Shannon indexes (P < 0.05, Fig. 2A) and Simpson indexes (P < 0.05, Fig. 2B), demonstrating that the microbiota diversity was significantly higher in the chemoresistant group than in the chemosensitive group. The richness of the chemoresistant group was slightly lower than that of the no chemotherapy resistance group, whereas the richness of the two groups did not differ significantly in the ACE indexes (P = 0.0809) and Chao1 indexes (P = 0.0709). Consistently, rarefaction curve analysis showed that the richness of chemoresistant OC patients tended to be lower than that of the control group (Fig. 2C). The rank abundance curve showed that the right tail of chemosensitive OC patients was longer, demonstrating a higher abundance and more uniform distribution than that of chemotherapy resistant patients (Fig. 2D). At the phylum level, we found that *Firmicutes, Proteobacteria* and* Bacteroidetes* are the main ones that occupy the dominant position. The dominant class are *Gammaproteobacteria, Bacteroidia* and *Clostridia*. The dominant species at the order level are *Enterobacteriales, Bacteroidales* and *Clostridiales*. The dominant species in the family level are *Enterobacteriaceae, Bacteroidaceae* and* Lachnospiraceae*. The dominant species in genus level were *unidentified*_*Enterobacteriaceae, Bacteroides* and* Faecalibacterium*.

**Figure 3:**
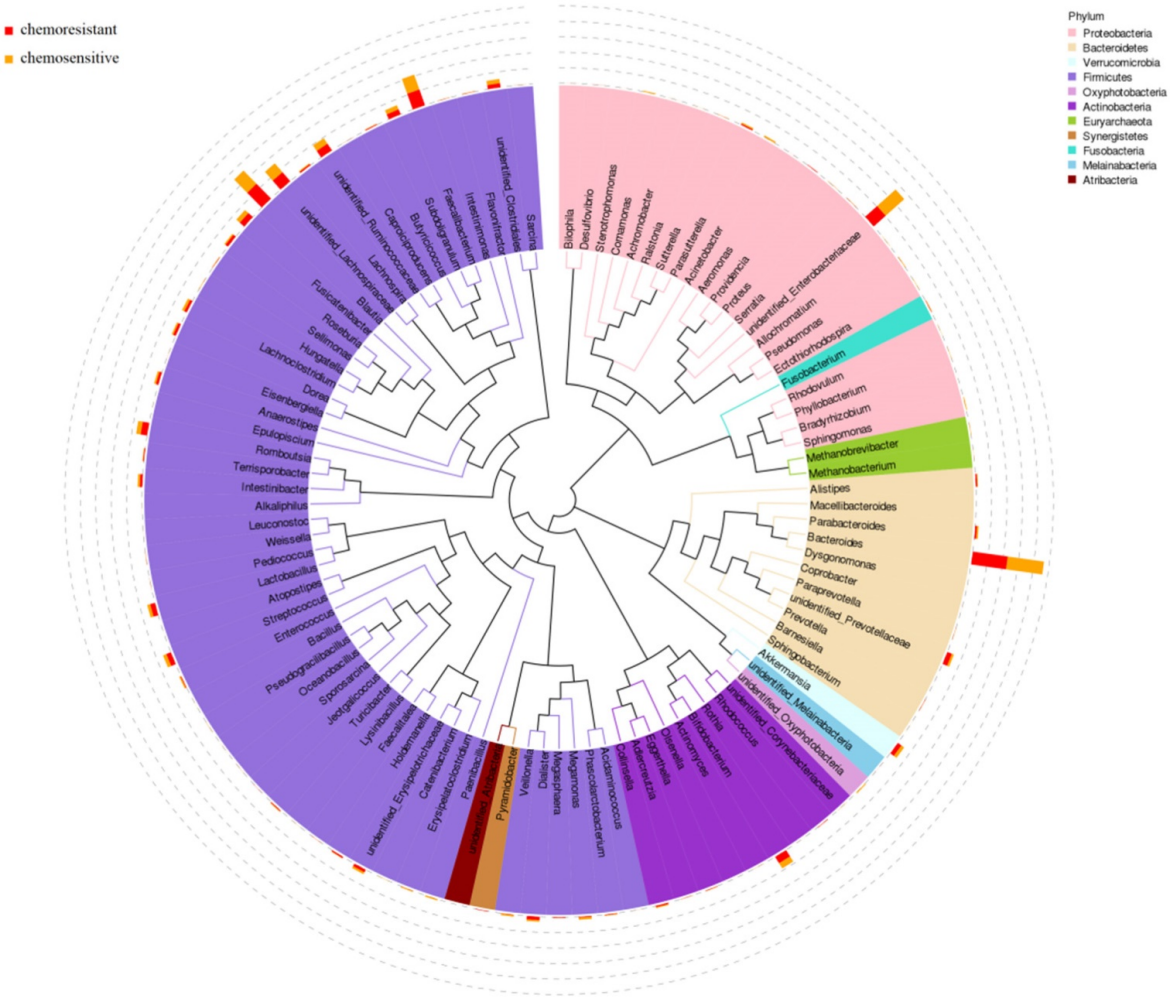
Phylogenetic tree of top 100 abundant species at genus level of ovarian cancer patients undergoing chemotherapy.

According to the results of 16S rRNA, the phylogenetic tree was constructed to represent the evolutionary relationship among various species. As shown in Fig. 3, *Firmicutes, Proteobacteria*, and *Bacteroidetes* phylum had higher abundance in both chemosensitive and chemoresistant OC patients. Among them, *Firmicutes* was the highest abundant phylum, which contains nearly half of all constructed evolutionary trees, fully demonstrating the richness and diversity of intestinal microbial community species. *Roseburia* also appears in the phylogenetic tree and belongs to *Firmicutes* phylum.

### Selection and implementation of machine learning methods

To select the best performing model for classification, we used RapidMiner to compare the performances of different machine learning approaches. The comparison results are as follows (Table 3). All models were implemented with the default parameters. The random forest model has the highest stability, and its prediction accuracy is 0.60, which is better than any single regression.

### Prediction of gut microbiota for the chemoresistant of OC

**Figure 4:**
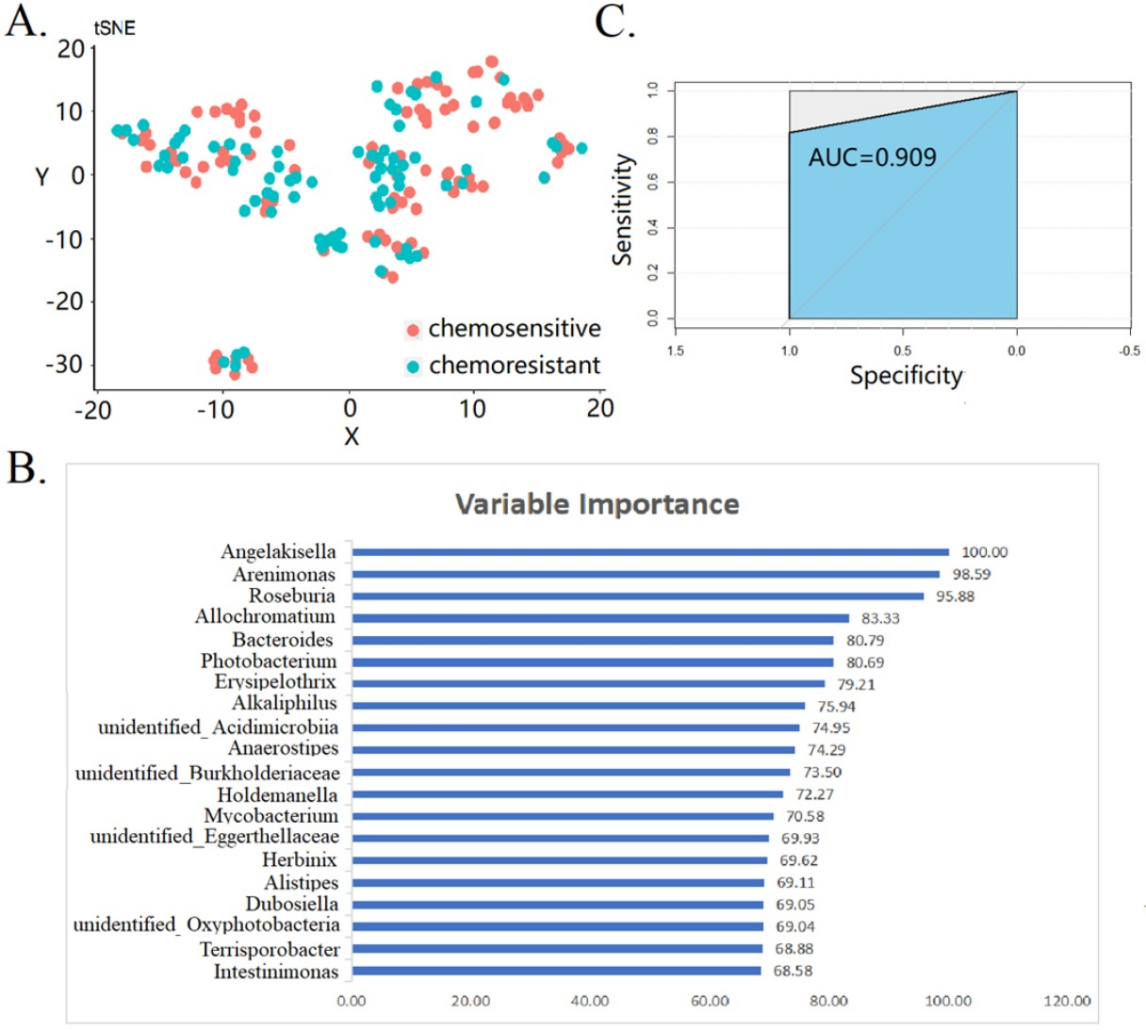
Prediction of gut microbiota for the chemoresistant of ovarian cancer patients. (A) tSNE dimensionality reduction analysis (B) top 20 variable importance in random forest model (C) receiver operating characteristic curve of random forest model.

Next, we attempted to predict the chemoresistant of OC by using gut microbiota and RF model. All the bacteria were treated with tSNE dimensionality reduction, and the uniformity reflected the sample quality control (Fig. 4A). The top 20 variable importance for RF-based prediction of chemoresistant of OC are shown in Fig. 4B. The mean decrease in gini index was used as a measure of variable importance. We found that* Angleakisella, Arenimonas,* and* Roseburia* have the top three importance in random forest model. The AUC values of the ROC curve was 0.909 (Fig. 4C). This proves that gut microbiota is related to chemotherapy resistance of and makes it possible to predict the effect of chemotherapy by gut microbiota. Findings were robust in the sensitivity analysis of patients with FIGO stage III-IV (data not shown).

## Discussion

To the best of our knowledge, this is the first study to investigate the differences in the gut microbiota between OC patients with and without chemotherapy resistance via 16S rRNA sequencing analysis. Due to the difficulty in identifying the key characteristic microbiota of the original relative abundance, machine learning methods can be used to find more characteristic relationships. Machine learning methods such as random forest model were used to construct the prediction of chemotherapy response of OC based on gut microbiota.

Analysis of the overall gut microbiota distribution showed that the gut microbiota diversity was higher in chemoresistant OC patients. Proteobacteria was the most abundant phylum in chemoresistant OC patients, and Firmicutes was found in chemosensitive OC patients. Among the nine machine learning methods, RF model had the highest prediction accuracy. Through RF model the relationship between gut microbiota and chemotherapy response of OC can be further established. Gut microbiota such as *Angelakisella*, *Arenimonas* and *Roseburia* was identified as a potential microbial biomarker for discriminating OC patients with chemoresistant OC patients from those chemosensitive.

There are few studies analyzing the relationship between the gut microbiota and cancer chemotherapy resistance. Choi et al. showed that the intestinal microbiota was related to chemotherapy resistance in colorectal cancer 32. These authors reported that enteropathogenic Escherichia coli induces macrophage inhibitory cytokine 1 to mediate the rapid growth and spread of intestinal cancers and causes colorectal cancer chemotherapy resistance. Zhang 33 and Yu 17 reported that high *Fusobacterium* nucleatum abundance is linked to colorectal cancer chemosensitivity. *Fusobacterium* nucleatum decreases the chemosensitivity of colorectal cancer to chemotherapy drugs, suggesting that the concentration of this bacterium may be an indicator of prognosis and an early warning of the risk of chemotherapy resistance. A recent study suggested that an unhealthy gut microbiota contributes to the aggressiveness and invasiveness of hormone receptor-positive breast cancer 34. Additionally, mouse experiments showed that the Salmonella enterica serotype Typhimurium inhibits the expression of P-glycoprotein, thereby increasing the chemosensitivity of cancer cells 35.

The present study had a similar research design by exploring the relationship between intestinal microbiota and chemotherapy resistance in OC 35. The involvement of the gut microbiota in the development and progression of OC has been reported previously. Zhou et al. 19 compared the microbiota between ovarian tissues and normal distal fallopian tube tissues, and showed that Proteobacteria and Firmicutes are bacteria associated with OC. This is consistent with the present study, as variations in Proteobacteria and Firmicutes were associated with OC with chemotherapy resistance.

The exact biological mechanisms linking gut microbiota to OC chemotherapeutic response are unknown. However, several potential mechanisms have been proposed. Cancer gene and epigenetic alterations associated with OC chemotherapeutic response have been widely reported 19. Recent studies in mice showed that the intestinal microbiota modulates immune responses, affecting chemotherapy and immunotherapy 38. Studies in humans showed that the adaptive immune system can modulate OC chemical sensitivity 39. *Roseburia* is a typical butyrate producing bacterium that plays a role in the treatment and prevention of atherosclerosis 40 and obesity-related diseases 41. *Roseburia* is increased in cervical cancer patients compared with healthy controls, indicating that it may serve as a novel potential biomarker for cervical cancer 42. A recent study showed that butyric acid, a short-chain fatty acid produced by dietary fiber fermented by the gut microbiota, can induce the expansion of Treg cells and the production of inflammatory cytokines, reduce tumor resistance, and inhibit the occurrence of colorectal cancer 43. The *Roseburia* genus and Fusobacterium nucleatum genus belong to the Clostridiales order. Fusobacterium nucleatum targets TLR4 and MYD88 innate immune signaling and specific microRNAs to activate the autophagy pathway and alter the chemotherapeutic response in colorectal cancer 17. Research on the resistance mechanism of *Roseburia* is limited. Additional basic research is warranted to explore the mechanism underlying the effect of *Roseburia* on OC chemotherapy resistance in the future. At present, there is no study on *Angelakisella*, and there are few studies on *Arenimonas*. *Angelakisella* are gram-negative bacilli with a very elongated shape 44. *Arenimonas* are gram-negative bacilli and usually found in natural soil and water environments 44.

The present study is the first to explore the relationship between the gut microbiota and OC patients with chemotherapy resistance. Furthermore, the sample size of our study was large. Machine learning methods such as random forest are powerful tools to predict the chemotherapy response of OC patients. All enrolled patients received optimally and maximally cytoreduced surgery and take the same chemotherapy regimens. The consistency in the enrolled patients is another strength of the study. The two groups were comparable in age, FIGO stage, and pathological typing, reducing the impact of confounding factors. The present study had some limitations. First, exercise and dietary information were not collected and analyzed. Exercise and diet can modify the gut microbiota, which in turn has a profound influence on health 47,48. However, the exercise and diet regimens of OC patients during chemotherapy are similar 49 and approximately 81% of OC patients do not meet the recommended physical activity guidelines. Second, although we excluded subjects who had taken antibiotics or probiotics in the month prior to the study, subjects who took other medicines for OC were not excluded for ethical reasons. Third, because of the case-control study design, we failed to determine the causal relationship between variation in the gut microbiota and chemotherapy resistance in OC. Whether changes in bacterial abundance are a causative factor for chemotherapy resistance or a response to the OC microenvironment needs to be further explored. Longitudinal studies should also be conducted to identify microbial biomarkers contributing to chemotherapy resistance in OC. Fourth, although the performance of internally validation of the RF model was good, this situation is not reality limited by the wave of intestinal changes as well as has high false positive. Additionally, we failed to carry out the external validation in the present study. Therefore, our findings should be interpreted with cautious. Future studies are warranted to validate our findings.

In summary, the present study showed that there are differences in the gut microbiota between OC patients with and without chemotherapy resistance. The diversity of the intestinal microbiota was higher in the chemoresistant group. RF model can predict the chemoresistance of OC based on gut microbiota. Further studies with better design and more detailed analysis are warranted in the future. Findings from these studies may provide the possibility of predicting the effect of chemotherapy treatment through intestinal flora in clinic, and guides doctors to choose different treatment schemes, so as to reduce the burden on patients.

## Figures and Tables

**Table 1:** Characteristics of ovarian cancer patients with or without chemotherapy resistance

	Total	Chemoresistant	Chemosensitive	*P* value
No. of case	174	77	97	
Age (Mean ± SD)	55.87±9.65	56.77±9.45	55.16±9.79	0.278
**FIGO stage (%)**				0.072
I	36 (20.7)	12 (15.6)	24 (24.7)	
II	8 (4.6)	1 (1.3)	7 (7.2)	
III	111 (63.8)	53 (68.8)	58 (59.8)	
IV	19 (10.9)	11 (14.3)	8 (8.2)	
**Pathological type (%)**				0.941
Serous	109 (62.6)	48 (62.3)	61 (62.9)	
Non-serous	65 (37.4)	29 (37.7)	36 (37.1)	

FIGO, International Federation of Gynecology and Obstetrics; SD, standard deviation.

**Table 2:** Comparison of phylotype coverage, richness, and diversity estimation of two groups according to 16S rRNA sequencing analysis

Group	No. of OUTs*	Good's (%)	Richness		Diversity	
			ACE	Chao1	Shannon	Simpson
Chemoresistant	2322	99.76	521	501	5.42	0.939
Chemosensitive	2776	99.75	547	522	5.19	0.925

*The operational taxonomic units (OTUs) were defined at the 97% similarity level.

**Table 3:** Performance comparison of different machine learning methods using RapidMiner

Model	Accuracy	Standard Deviation	Gains
Naive Bayes	0.56	0.1	0.0
Generalized Linear Model	0.58	0.1	4.0
Logistic Regression	0.56	0.1	0.0
Fast Large Margin	0.50	0.1	-6.0
Deep Learning	0.56	0.1	0.0
Decision Tree	0.50	0.1	-4.0
Random Forest	0.60	0.1	4.0
Gradient Boosted Trees	0.54	0.1	-4.0
Support Vector Machine	0.58	0.1	2.0
